# Use of antipsychotics and benzodiazepines in patients with psychiatric emergencies: Results of an observational trial

**DOI:** 10.1186/1471-244X-8-61

**Published:** 2008-07-22

**Authors:** Stefan Wilhelm, Alexander Schacht, Thomas Wagner

**Affiliations:** 1 Medical Department, Lilly Deutschland GmbH, Saalburgstraße 153, Bad Homburg, Germany

## Abstract

**Background:**

Conventional antipsychotics augmented with benzodiazepines have been the standard acute treatment for psychiatric emergencies for more than 50 years. The inability of patients to give informed consent limits randomised, controlled studies. This observational study on immediate therapy for aggression and impulse control in acutely agitated patients (IMPULSE) evaluated the short-term effectiveness and tolerability of atypical and typical antipsychotic medications (AP) in a non-interventional setting.

**Methods:**

This was a comparative, non-randomised, prospective, open-label, observational study. Treatment over the first 5 days was classified according to whether any olanzapine, risperidone, or haloperidol was included or not. Documentations (PANSS-excited component, CGI-aggression, CGI-suicidality, tranquilisation score) were at baseline (day 1) and days 2–6 after start of AP.

**Results:**

During the short treatment-period, PANSS-EC and CGI-aggression scores improved in all cohorts. 68.7% of patients treated with olanzapine, 72.2% of patients treated with risperidone, and 83.3% of patients treated with haloperidol received concomitant benzodiazepines (haloperidol vs. non-haloperidol: p < 0.001). More patients treated with olanzapine (73.8%) were fully alert according to a tranquilisation score and active at day 2 than patients treated with risperidone (57.1%) or haloperidol (58.0%).

**Conclusion:**

Current medication practices for immediate aggression control are effective with positive results present within a few days. In this study, concomitant benzodiazepine use was significantly more frequent in patients receiving haloperidol.

## Background

Behavioural emergencies occur in approximately 1% to 20% of psychiatric patients admitted to hospitals and necessitate physical restraint, seclusion and involuntary (parenteral) medication [1-3]. Serious injuries to both patients and staff can occur. However, the inability of acutely agitated patients to give informed consent limits the possibility to conduct randomised, controlled studies evaluating the effectiveness of different treatment options [2]. A non-interventional, observational study can overcome these limitations.

For more than 50 years, conventional antipsychotics augmented with benzodiazepines have been the standard treatment [4,5]. The most important side-effects of acute typical antipsychotic treatment are acute dystonic reactions, which can be both alarming and painful, but can be avoided by using second-generation (atypical) antipsychotics [6]. The objectives of this observational study were to document immediate therapy (use of antipsychotic medication and extent of concomitant benzodiazepine use) for aggression and impulse control in acutely agitated patients, and to evaluate the short-term effectiveness and tolerability of atypical and typical antipsychotics (AP) in this non-interventional setting (IMPULSE study).

## Methods

### Study design and population

The IMPULSE study was a non-randomised, non-interventional, open-label, prospective, multi-centre observational study in an unselected sample of patients with behavioural emergencies. The study description asked for patients with schizophrenia, meeting the following criteria for participation: adult patients (≥ 18 years) newly admitted to a psychiatric or forensic hospital, presenting with agitation with or without aggression and requiring antipsychotic treatment. Patients were recruited in Germany between July 2003 and October 2004. Because this was a non-interventional, observational study according to German Drug Law (AMG) §67 (6), explicit ethical approval by an institutional review board or written patient consent was not required. The German Federal Institute for Drugs and Medical Devices ("Bundesinstitut für Arzneimittel und Medizinprodukte", BfArM) and the German Statutory Health Insurance of Physicians ("Kassenärztliche Bundesvereinigung", KBV) were notified about this observational study prior to the start of data collection. Decisions on patient recruitment, the antipsychotic treatment given and use or non-use of benzodiazepines were completely at the discretion of the investigator. Due to the lack of randomisation and pre-defined recruitment rules, patients receiving olanzapine were over-represented when compared to the olanzapine market share (IMS Health, approximately 30%) of antipsychotic agents in Germany during the recruitment period in 2003–2004. Moreover, the investigators also recruited patients with psychiatric disorders other than schizophrenia.

### Assessments and measures

Assessments were carried out daily, at baseline (day 1) and the following 5 days after initial documentation (days 2–6). Diagnoses were documented according to the International Classification of Diseases, 10th revision (ICD-10) [7]. The presence of comorbid substance use disorder (SUD) was assessed according to ICD-10 criteria [7].

Psychiatric emergencies were categorised as follows: (i) Self-endangering behaviour included acute suicidal thoughts or plans, compulsatory admission because of suicidality as well as self-injuring behaviour; (ii) Third party endangering was defined to be present if patients presented with one or more of the following symptoms: intimidating behaviour, aggression to property, demeaning or hostile verbal behaviour, or aggression to persons; (iii) Agitation was defined according to Lindenmayer [8] as a state of poorly organised and aimless psychomotor activity including symptoms such as motor restlessness, heightened responsiveness to external or internal stimuli, irritability, and/or decreased sleep.

Patients' antipsychotic treatment was categorised as including any olanzapine or not (Olz or non-Olz), including any risperidone or not (Ris or non-Ris), and including any haloperidol or not (Hal or non-Hal). The Olz, Ris, and Hal cohorts thus overlap, because each cohort included all patients who received the respective drug in any amount and at any time throughout the 5-day study period.

Benzodiazepine dosages are presented as cumulative dose during the complete study period, expressed as mg diazepam equivalents. Those were calculated based on the data published by Poser and Poser in 1996 [9].

The following instruments were completed at each visit: Agitation was measured using the Excited Component of the Positive and Negative Syndrome Scale (PANSS) [10,11]. The PANSS-EC includes the following 5 items from the PANSS, rated from 1 (not present) to 7 (extremely severe): poor impulse control, tension, hostility, uncooperativeness, and excitement (score range from 5 to 35; mean scores ≥ 20 clinically correspond with severe agitation) [12]. PANSS-EC response rates were also calculated, with response defined as ≥ 40% reduction from baseline in PANSS-EC scores on day 6. Additional instruments included the Clinical Global Impression Severity of Illness – Aggression (CGI-A) and CGI-Suicidality (CGI-SS) Scales. The standard CGI scale [13,14] has been adapted to measure severity of specific syndromes, including aggression in patients with psychiatric disorders (CGI-A) [15-17] and suicidality (CGI-SS) [18]. 5-point likert scales were used for the CGI-A (1 = not at all aggressive, 5 = aggressive behaviour) and the CGI-SS (1 = not at all suicidal, 5 = attempted suicide). Finally, a single-item, 5-point tranquilisation scale (1 = fully alert and active, 5 = deeply asleep) was used to assess alertness of the patients [19]. Treatment tolerability was assessed by adverse event recording at each visit. Adverse events were coded using the MedDRA classification system

### Statistics

Based on market research data for the clinical setting from January 2002 through January 2003, it was expected that cohorts would contain at least 75 patients and up to approximately 300 patients. Based on clinical trial data it was assumed that 43% to 53% of patients would receive benzodiazepine co-medication, and the corresponding two-sided 95% confidence intervals for these rates would be approximately 11.2% (N = 75) and 5.6% (N = 300). The standard deviations (SD) of mean daily benzodiazepine doses ranged from 3.2 mg to 5.8 mg in clinical trial data. However, compared to clinical trials, a larger standard deviation of daily benzodiazepine doses must be assumed due to the naturalistic setting of the present study. Assuming a SD of 7 mg leads to two-sided 95% confidence intervals with a distance from mean to limit of 1.58 mg (N = 75) and 0.79 mg (N = 300). Therefore, the planned total number of 500 patients was considered sufficient to observe different therapeutic responses in a population of impulsive/aggressive patients with psychiatric emergencies.

Due to the naturalistic and observational design of the study, a specific primary outcome measure as required for an interventional clinical trial was not defined; all analyses were exploratory only. No confirmatory statistical tests were performed, but 95% confidence intervals and p values were calculated for exploratory treatment group comparisons. Chi-square tests were used for categorical data, t-tests with pooled variance were used for continuous data. The odds ratios and the respective confidence intervals for the use of benzodiazepines at any time were calculated by cohort using the logistic regression technique. Except for the PANSS-EC, all parameters were analyzed on a categorical basis whereby each item at each time point was analyzed descriptively.

## Results

### Patient characteristics and disposition

A total of 558 patients with acute behavioural emergencies were enrolled at 102 participating centres (median 5 patients per centre). Patient demographics are presented in Table 1. More than 90% of patients (n = 523, 93.7%) were assessed at psychiatric hospitals (forensic hospital 2.3%, missing data 3.9%). 390 patients (69.9%) received at least one dose of olanzapine during the 5-day study period and were therefore included in the olanzapine (Olz) cohort. Of these, 177 patients suffered from schizophrenia (n = 147) or manic episodes (n = 30), and were therefore treated in-label. The remaining 30.1% of patients were grouped in the non-olanzapine (non-Olz) cohort. 132 patients (23.7%) received any haloperidol (Hal), and 72 patients (12.9%) received any risperidone (Ris).

**Table 1:** Patient demographics, diagnosis and characteristics by treatment cohort

**Parameter [statistic]**	**All patients**	**Olanzapine vs. non-olanzapine**			**Risperidone vs. non-risperidone**			**Haloperidol vs. non-haloperidol**		
				
		**OLZ**	**Non-OLZ**		**RIS**	**Non-RIS**		**HAL**	**Non-HAL**	
	**(N = 558)**	**(N = 390)**	**(N = 168)**	**p-value**	**(N = 72)**	**(N = 486)**	**p-value**	**(N = 132)**	**(N = 426)**	**p-value**
**Patient demographics**										
Gender (male), n (%)	353 (63.3)	237 (60.8)	116 (69.0)	0.068	46 (63.9)	307 (63.2)	0.923	93 (70.5)	260 (61.0)	0.053
Age, median (range), y	38 (18–93)	37 (18–93)	39 (19–84)	0.068	40 (19–87)	38 (18–93)	0.081	39 (18–93)	38 (18–90)	0.223
**Primary psychiatric diagnosis**^a^ (ranked according to overall frequency)^b^										
Schizophrenia spectrum disorders (F20–F29), n (%)	330 (59.1)	215 (55.1)	115 (68.5)	0.003	50 (69.4)	280 (57.6)	0.057	92 (69.7)	238 (55.9)	0.005
Disorders due to substance use (F10–F19), n (%)	98 (17.6)	69 (17.7)	29 (17.3)	0.902	7 (9.7)	91 (18.7)	0.061	23 (17.4)	75 (17.6)	0.962
Mood (affective) disorders (F30–F39), n (%)	88 (15.8)	80 (20.5)	8 (4.8)	< .001	4 (5.6)	84 (17.3)	0.011	15 (11.4)	73 (17.1)	0.112
Disorders of adult personality and behaviour (F60–F69), n (%)	84 (15.1)	67 (17.2)	17 (10.1)	0.032	10 (13.9)	74 (15.2)	0.767	4 (3.0)	80 (18.8)	< .001
Organic, including symptomatic mental disorders (F00–F09), n (%)	69 (12.4)	39 (10.0)	30 (17.9)	0.010	14 (19.4)	55 (11.3)	0.051	19 (14.4)	50 (11.7)	0.418
Other^c^, N (%)	57 (10.2)	43 (11.0)	14 (8.3)	0.335	8 (11.1)	49 (10.1)	0.788	5 (3.8)	52 (12.2)	0.005

**Co-morbid substance use disorder (SUD) at baseline**^b^										
Nicotine, n (%)	241 (43.2)	172 (44.1)	69 (41.1)	0.507	28 (38.9)	213 (43.8)	0.430	50 (37.9)	191 (44.8)	0.159
Alcohol, n (%)	103 (18.5)	68 (17.4)	35 (20.8)	0.343	11 (15.3)	92 (18.9)	0.456	25 (18.9)	78 (18.3)	0.871
Illicit drugs, n (%)	83 (14.9)	59 (15.1)	24 (14.3)	0.798	9 (12.5)	74 (15.2)	0.544	24 (18.2)	59 (13.8)	0.222

**Type of behavioural emergency at baseline**^b^										
Self-endangering, n (%)	264 (47.3)	186 (47.7)	78 (46.4)	0.784	28 (38.9)	236 (48.6)	0.125	68 (51.5)	196 (46.0)	0.268
Third party endangering, n (%)	393 (70.4)	266 (68.2)	127 (75.6)	0.079	57 (79.2)	336 (69.1)	0.082	108 (81.8)	285 (66.9)	0.001
Agitation, n (%)	408 (73.1)	294 (75.4)	114 (67.9)	0.066	53 (73.6)	355 (73.0)	0.919	98 (74.2)	310 (72.8)	0.739
**Type of admission**										
Compulsory admission, n (%)	238 (42.7)	159 (40.8)	79 (47.0)	0.171	32 (44.4)	206 (42.4)	0.742	78 (59.1)	160 (37.6)	< .001
**Premature discontinuation, n (%)**	16 (2.9)	9 (2.3)	7 (4.2)	0.223	3 (4.2)	13 (2.7)	0.465	6 (4.5)	10 (2.3)	0.188

^a ^ICD-10 diagnostic groups are listed according to the frequency among all patients.

^b ^Patients may have received more than one ICD-10 diagnosis or more than one behavioural disturbance.

^c ^Behavioural and emotional disorders with onset usually occurring in childhood and adolescence (F90–F98); behavioural syndromes associated with physiological disturbances and physical factors (F50–F59); neurotic, stress-related and somatoform disorders (F40–F48); and mental retardation (F70–F79)

Abbreviations: OLZ = olanzapine, RIS = risperidone, HAL = haloperidol.

Oral antipsychotic medication was used in the majority of cases, but parenteral (intravenous or intramuscular) acute or depot formulations were used as well. In the haloperidol group (n = 132), 46 patients (34.9%) received intravenous, 16 patients (12.1%) intramuscular and 7 patients (5.3%) depot injections. In the olanzapine group (n = 389 with data available), 27 patients (6.9%) received an intramuscular preparation. In the risperidone group (n = 72), 5 patients (6.9%) each received intramuscular and depot injections.

Most patients were diagnosed with schizophrenia spectrum disorders (n = 330, 59.1%). Other diagnoses and the various types of psychiatric emergencies are presented in Table 1. At baseline, only a small proportion of patients (n = 9, 1.6%) had received prior treatment with anticholinergics.

Almost all patients completed the 5 days study period (97.4%); 1.2% of patients were lost to follow-up, 0.6% discontinued due to adverse events, and 0.8% discontinued due to other reasons.

Overall, 38.4% of patients had received prior antipsychotic treatment (Table 2). Patients in the olanzapine cohort were less frequently pre-treated with antipsychotics than patients receiving no olanzapine (33.3% vs. 50.0%, p < 0.001), while patients receiving risperidone were more frequently pre-treated than those without risperidone (52.8% vs. 36.2%, p = 0.007), and no difference was identified for the haloperidol versus non-haloperidol cohort. No significant treatment group differences were identified for prior benzodiazepine, mood stabilizer, or antidepressive treatment.

**Table 2:** Treatment variables by treatment cohort

**Parameter [statistic]**	**All patients**	**Olanzapine vs. non-olanzapine**			**Risperidone vs. non-risperidone**			**Haloperidol vs. non-haloperidol**		
				
		**OLZ**	**Non-OLZ**		**RIS**	**Non-RIS**		**HAL**	**Non-HAL**	
	**(N = 558)**	**(N = 390)**	**(N = 168)**	**p-value**	**(N = 72)**	**(N = 486)**	**p-value**	**(N = 132)**	**(N = 426)**	**p-value**
**Previous pharmacological treatment**^a^										
Any previous medication n (%)	291 (52.2)	191 (49.0)	100 (59.5)	0.022	45 (62.5)	246 (50.6)	0.060	64 (48.5)	227 (53.3)	0.335
Any antipsychotic n (%)	214 (38.4)	130 (33.3)	84 (50.0)	< .001	38 (52.8)	176 (36.2)	0.007	52 (39.4)	162 (38.0)	0.778
*Olanzapine, n (%)*	*52 (9.3)*	*48 (12.3)*	*4 (2.4)*		*5 (6.9)*	*47 (9.7)*		*13 (9.8)*	*39 (9.2)*	
*Risperidone, n (%)*	*50 (9.0)*	*26 (6.7)*	*24 (14.3)*		*30 (41.7)*	*20 (4.1)*		*11 (8.3)*	*39 (9.2)*	
*Haloperidol, n (%)*	*45 (8.1)*	*30 (7.7)*	*15 (8.9)*		*5 (6.9)*	*40 (8.2)*		*25 (18.9)*	*20 (4.7)*	
*Quetiapine, n (%)*	*19 (3.4)*	*6 (1.5)*	*13 (7.7)*		*0 (0.0)*	*19 (3.9)*		*4 (3.0)*	*15 (3.5)*	
Any benzodiazepine n (%)	106 (19.0)	75 (19.2)	31 (18.5)	0.830	10 (13.9)	96 (19.8)	0.236	22 (16.7)	84 (19.7)	0.435
*Lorazepam, n (%)*	*51 (9.1)*	*36 (9.2)*	*15 (8.9)*		*6 (8.3)*	*45 (9.3)*		*14 (10.6)*	*37 (8.7)*	
*Diazepam, n (%)*	*43 (7.7)*	*29 (7.4)*	*14 (8.3)*		*3 (4.2)*	*40 (8.2)*		*7 (5.3)*	*36 (8.5)*	
Mood stabilizer n (%)	67 (12.0)	48 (12.3)	19 (11.3)	0.939	7 (9.7)	60 (12.3)	0.568	11 (8.3)	56 (13.1)	0.141
Antidepressants n (%)	64 (11.5)	47 (12.1)	17 (10.1)	0.679	8 (11.1)	56 (11.5)	0.976	9 (6.8)	55 (12.9)	0.056

**Antipsychotic monotherapy** **n (%)**	227 (40.7)	183 (46.9)	44 (26.2)	< .001	13 (18.1)	214 (44.0)	< .001	11 (8.3)	216 (50.7)	< .001
**Concomitant benzodiazepine use**										
Any benzodiazepine use n (%) [95% CI (%)]	389 (69.7) [65.7–73.5]	268 (68.7) [63.9–73.3]	121 (72.0) [64.6–78.7]	0.436	52 (72.2) [60.4–82.1]	337 (69.3) [65.0–73.4]	0.620	110 (83.3) [75.9–89.3]	279 (65.5) [60.8–70.0]	< .001
*Lorazepam, n (%)*	*248 (44.4)*	*177 (45.4)*	*71 (42.3)*		*35 (48.6)*	*213 (43.8)*		*65 (49.2)*	*183 (43.0)*	
*Diazepam, n (%)*	*175 (31.4)*	*109 (27.9)*	*66 (39.3)*		*29 (40.3)*	*146 (30.0)*		*75 (56.8)*	*100 (23.5)*	
*Other, n (%)*	*64 (11.5)*	*38 (9.7)*	*26 (15.5)*		*12 (16.7)*	*52 (10.7)*		*12 (9.1)*	*52 (12.2)*	
Benzodiazepine dose^b^ mean (SD), mg	81.3 (58.1)	77.9 (52.5)	89.0 (68.5)	0.081	86.4 (72.5)	80.6 (55.6)	0.497	76.4 (50.6)	83.3 (60.8)	0.296

^a ^Patients may have received more than one previous pharmacological medication.

^b ^Cumulative dose over the complete study period in mg diazepam equivalents.

Abbreviations: OLZ = olanzapine, RIS = risperidone, HAL = haloperidol.

During the study, the proportion of patients receiving antipsychotic monotherapy was significantly higher in the olanzapine than in the non-olanzapine cohort (46.9% vs. 26.2%, p < 0.001), but significantly lower in the risperidone vs. the non-risperidone and in the haloperidol vs. non-haloperidol cohorts (p < 0.001, Table 2).

Overall, 389 patients (69.7%) received concomitant benzodiazepine treatment, mostly lorazepam (*n *= 248, 44.4%) or diazepam (*n *= 175, 31.4%). Table 2 presents benzodiazepine treatment by treatment cohort. Mean cumulative doses (in diazepam equivalents) over 5 days tended to be lower in the olanzapine than in the non-olanzapine cohort (77.9 mg vs. 89.0 mg, p = 0.081), no differences were identified for the other cohorts. In the olanzapine cohort, 68.7% of patients received any benzodiazepine treatment, as compared to 72.2% in the risperidone and 83.3% in the haloperidol cohorts. Patients in the haloperidol cohort were treated with benzodiazepines more frequently than those in the non-haloperidol cohort: the odds-ratio for receiving any concomitant benzodiazepines was 2.33 (95% CI: 1.39 – 3.85) for haloperidol vs. non-haloperidol, as compared to 0.91 (95% CI: 0.60 – 1.37) for olanzapine vs. non-olanzapine and 1.12 (95% CI: 0.64 – 2.00) for risperidone vs. non-risperidone.

### Short-term effectiveness

#### PANSS-EC

**Figure 1:**
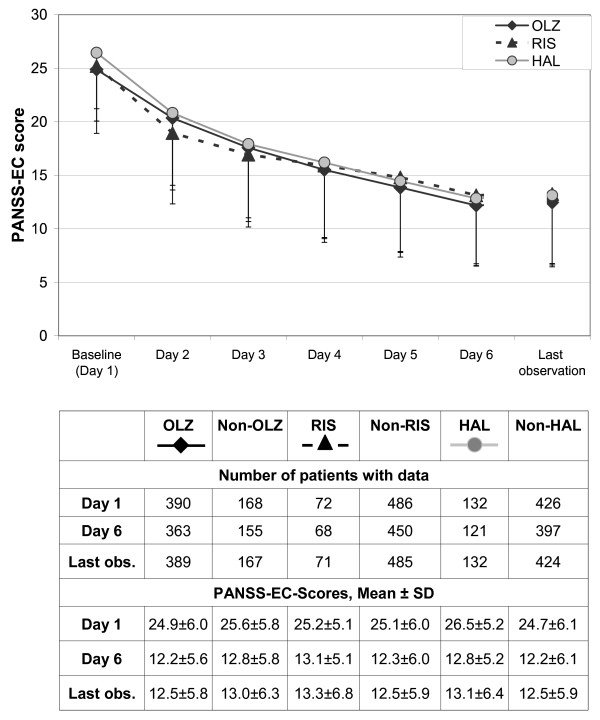
**Course of PANSS-excited component (PANSS-EC) scores (means and standard deviations) by antipsychotic treatment cohort**. Note: Patients in the olanzapine, risperidone and haloperidol cohorts may overlap because all olanzapine, risperidone, or haloperidol patients are included who received at least one dose of the respective drug. Abbreviations: OLZ = olanzapine, RIS = risperidone, HAL = haloperidol. Last obs = last observation.

Figure 1 presents the course of the PANSS-EC score from baseline to endpoint for the olanzapine, risperidone and haloperidol cohorts. At baseline, mean PANSS-EC scores were 25 or higher, corresponding to a clinically severe agitation syndrome. Up to the last day, PANSS-EC scores decreased significantly over time in all treatment cohorts.

The percentage of patients classified as responders (PANSS-EC ≥ 40% reduced) did not differ significantly between treatment cohorts (Olz vs. non-Olz 70.0% vs. 66.7%, Ris vs. non-Ris 69.4% vs. 68.9%, Hal vs. non-Hal 71.2% vs. 68.3%, p > 0.5 for all comparisons).

#### CGI-aggression and CGI-suicidality scores

**Figure 2:**
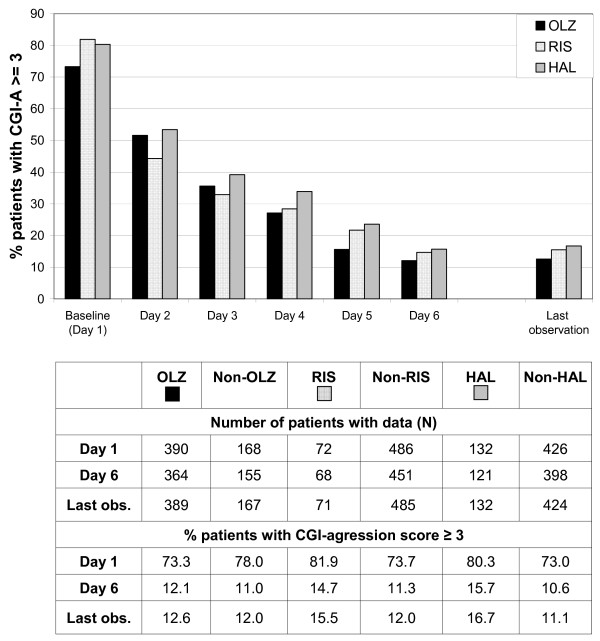
**CGI-agression (CGI-A) scores by antipsychotic treatment cohort; percentage of patients with CGI-A scores ≥ 3**. Note: Patients in the olanzapine, risperidone and haloperidol cohorts may overlap because all olanzapine, risperidone, or haloperidol patients are included who received at least one dose of the respective drug. Abbreviations: OLZ = olanzapine, RIS = risperidone, HAL = haloperidol.Figure legend text.

The proportion of patients who had at least a moderate level of aggression (CGI-A score ≥ 3) decreased progressively over the 5-day observation period, with no marked differences between treatment groups (Figure 2). However, it must be noted that the olanzapine cohort tended to have lower CGI-A ratings at baseline (CGI-A ≥ 3: Olz 73.3%, Ris 81.9%, Hal 80.3%).

**Figure 3:**
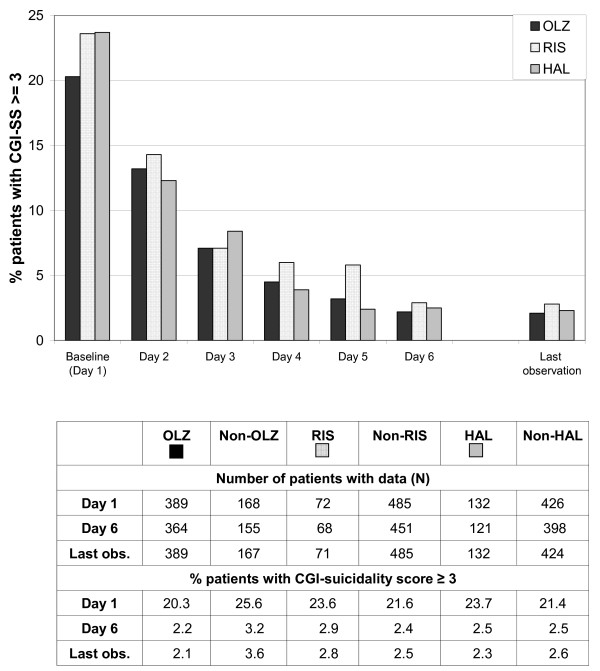
**CGI-suicidality (CGI-SS) scores by antipsychotic treatment cohort; percentage of patients with CGI-SS scores ≥ 3**. Note: Patients in the olanzapine, risperidone and haloperidol cohorts may overlap because all olanzapine, risperidone, or haloperidol patients are included who received at least one dose of the respective drug. Abbreviations: OLZ = olanzapine, RIS = risperidone, HAL = haloperidol.

The proportion of patients rated at least moderately suicidal (CGI-SS score ≥ 3) also decreased over the 5-day observation period in all treatment cohorts (Figure 3). Again, it must be noted that the olanzapine cohort tended to have lower CGI-SS ratings at baseline (CGI-SS ≥ 3: Olz 20.3%, Ris 23.6%, Hal 23.7%).

### Tranquilisation score

**Figure 4:**
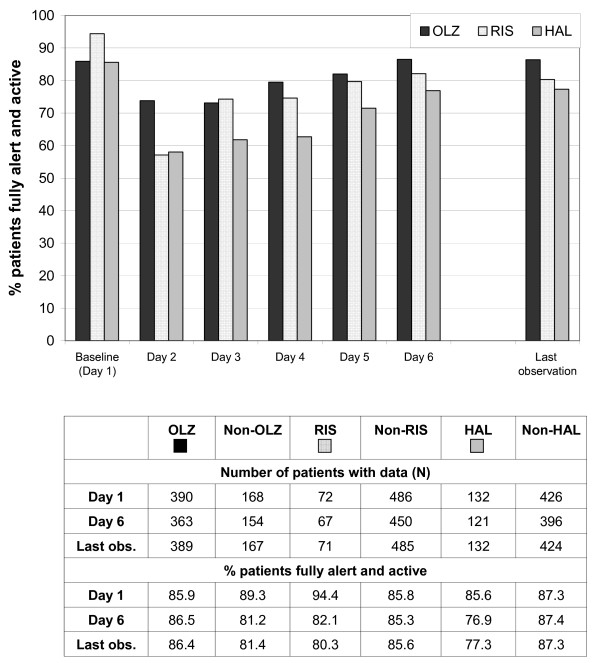
**Tranquilisation score by antipsychotic treatment cohort; percentage of patients fully alert and active**. Note: Patients in the olanzapine, risperidone and haloperidol cohorts may overlap because all olanzapine, risperidone, or haloperidol patients are included who received at least one dose of the respective drug. Abbreviations: OLZ = olanzapine, RIS = risperidone, HAL = haloperidol.

In all treatment cohorts, the percentage of patients rated as "fully alert and active" (tranquilisation score = 1) decreased from baseline to day 2 (Figure 4). However, the proportion of patients who were still fully alert and active on day 2 was markedly higher in the olanzapine cohort (73.1%) than in the risperidone (55.6%) or haloperidol (57.6%) cohorts. Over the remaining days of the observation, the percentage of fully alert and active patients gradually increased again in all cohorts, and this percentage remained highest in the olanzapine cohort on all post-baseline days (Figure 4).

### Tolerability

Treatment-emergent adverse events were reported in 37 patients (6.6%) of patients overall, the overall adverse event rate was in the same order of magnitude for all treatment cohorts (Table 3). The frequencies of specific adverse events reported for the different treatment cohorts are of limited validity due to the small numbers. However, the adverse events reported most frequently in the olanzapine cohort were sedation, dizziness and weight gain (n = 3, 0.8% each). Dyskinesia (n = 3, 2.3%), extrapyramidal disorder, sedation, and dizziness (n = 2, 1.5% each) were reported most in the haloperidol cohort. In the risperidone cohort, no adverse event was reported more than once. Serious adverse events were reported in four patients; the investigator considered them as possibly related to the primary AP medication in 2 patients (delirium due to prescribed overdose [olanzapine 30–35 mg/d for 2 days], related to olanzapine treatment; convulsions of moderate severity, related to haloperidol treatment). Two patients discontinued their AP due to adverse events (olanzapine: the serious delirium event; risperidone: non-serious dystonia).

**Table 3:** Adverse events

**Event, MedDRA preferred term**	**All patients**	**Olanzapine **	**Non-Olanzapine **	**Risperidone **	**Non-Risperidone **	**Haloperidol **	**Non-Haloperidol **
	**(N = 558)**	**(N = 390)**	**(M = 168)**	**(N = 72)**	**(N = 486)**	**(N = 132)**	**(N = 426)**
**Treatment-emergent adverse events, n (%)**	37 (6.6)	24 (6.2)	13 (7.7)	8 (11.1)	29 (6.0)	13 (9.8)	24 (5.6)
Dyskinesia^a^, n (%)	4^a^ (0.7)	2 (0.5)	2 (1.2)	0 (0.0)	4 (0.8)	3 (2.3)	1 (0.2)
Extrapyramidal disorder, n (%)	4 (0.7)	1 (0.3)	3 (1.8)	1 (1.4)	3 (0.6)	2 (1.5)	2 (0.5)
Sedation, n (%)	4 (0.7)	3 (0.8)	1 (0.6)	1 (1.4)	3 (0.6)	2 (1.5)	2 (0.5)
Dizziness postural, n (%)	3 (0.5)	3 (0.8)	0 (0.0)	0 (0.0)	3 (0.6)	2 (1.5)	1 (0.2)
Weight increased, n (%)	3 (0.5)	3 (0.8)	0 (0.0)	1 (1.4)	2 (0.4)	0 (0.0)	3 (0.7)
Akathisia, n (%)	2 (0.4)	2 (0.5)	0 (0.0)	1 (1.4)	1 (0.2)	0 (0.0)	2 (0.5)
Oculogyric crisis, n (%)	2 (0.4)	2 (0.5)	0 (0.0)	1 (1.4)	1 (0.2)	0 (0.0)	2 (0.5)
Salivary hypersecretion, n (%)	2 (0.4)	1 (0.3)	1 (0.6)	1 (1.4)	1 (0.2)	0 (0.0)	2 (0.5)
							
Adverse events considered related to primary AP medication, n (%)	33 (5.9)	21 (5.4)	12 (7.1)	8 (11.1)	25 (5.1)	12 (9.1)	21 (4.9)
Serious adverse events, n (%)	4 (0.7)	2 (0.5)	2 (1.2)	0 (0.0)	4 (0.8)	3 (2.3)	1 (0.2)
Serious adverse events considered related to primary AP medication, n (%)	2 (0.4)	1 (0.3)	1 (0.6)	0 (0.0)	2 (0.4)	2 (1.5)	0 (0.0)
Clinically significant adverse events resulting in discontinuation, n (%)	2 (0.4)	1 (0.3)	1 (0.6)	1 (1.4)	1 (0.2)	0 (0.0)	2 (0.5)

Only preferred terms occurring in at least 2 patients are listed.

Patients may be included in more than one category of event or preferred term.

^a ^Includes the following original terms as reported by physicians: early dyskinesias (n = 3), perioral dyskinesia (n = 1).

## Discussion

More than 4 million people are admitted to emergency treatment facilities annually in the USA, in an acute condition requiring immediate treatment to control aggressive and impulsive behaviours [20]. Overall, these acutely aggressive or impulsive patients account for more than 5% of all admissions to emergency treatment facilities in the USA [20]. In a 1995 study of emergency medical calls in Hamburg, Germany, the treatment of aggressive or impulsive patients was found to account for nearly 10% of all calls for emergency physicians, and was the third most common reason for such calls [21]. Given that emergency treatment of such patients is so common and so urgent, it is disappointing and surprising that no generally agreed-upon guidelines exist for the emergency treatment of these patients [22-24]. However, a number of factors have contributed to the dearth of controlled clinical studies for the treatment of acutely aggressive or impulsive patients.

The psychiatric sources of these behaviours, as well as the physical and mental status of these patients at admission, are extremely varied and are often unknown to the treating physician. For example, such patients may have a history of schizophrenia (accounting for approximately 21% of visits to emergency treatment facilities in the USA [22]), bipolar disorder, personality disorder, or other organic disorder, and this population also typically includes users (and abusers) of legal or illegal psychoactive substances [25,26]. In our study, this fact is also reflected by a high percentage of off-label use, e.g. less than 50% of the patients within the olanzapine cohort were treated according to the marketed indication. Also, the necessity of treating these acutely aggressive and impulsive patients immediately, often without time for a psychiatric or physical diagnosis, creates a need for antipsychotic medications that are rapidly effective, and safe for an extremely disparate group of patients which may range from teenage drug abusers to patients with Alzheimer's Disease.

Due to this urgency, combined with only scarce evidence from controlled clinical studies, treating physicians generally rely upon older established medications (such as typical APs with or without concomitant benzodiazepines) that may differ from more recently-introduced medications such as the atypical APs. Some controlled trials have evaluated typical APs for emergency treatment, underlining their effectiveness. In one controlled, double-blind study, an intramuscular injection of haloperidol and lorazepam was more rapid in achieving emergency tranquilisation than either haloperidol or lorazepam alone [5]. Two large pragmatic, randomised studies conducted in India and Brazil have evaluated intramuscular haloperidol-promethazine combination treatment, a standard treatment for patients in these countries, versus a rapid-acting intramuscular benzodiazepine (midazolam, lorazepam) [27,28]. Both interventions were effective in controlling agitated behaviour; however, midazolam was more rapidly sedating than haloperidol-promethazine in one study [27], while lorazepam was acting slower than haloperidol-promethazine in the other study [28]. One recent double-blind controlled study showed greater efficacy of the atypicals clozapine and olanzapine versus haloperidol in reducing aggressive behaviour in long-term management of inpatients [29]. However, carefully-controlled clinical studies of atypicals versus typicals for emergency treatment are scarce, but a number of comprehensive literature reviews have concluded that the atypical APs are at least as effective and generally have better tolerability than the typical APs administered with or without benzodiazepines [3,22,23,30]. For example, in a double-blind comparison of olanzapine versus the benzodiazepine lorazepam for treatment of acute psychiatric episodes, olanzapine treatment was found equally effective and with a better tolerability profile, particularly with respect to the incidence of EPS [31,32].

Another reason for the paucity of controlled clinical trials in patients with acute aggressive or impulsive behaviour is that until recently most atypical APs were not available as intramuscular formulations [3], which many physicians prefer for acute treatment of aggressive patients. However, intramuscular formulations of ziprasidone and olanzapine are now available, and results from controlled studies indicated that these are equally effective and fast-acting as the commonly-used intramuscular formulations of APs [30-33]. In addition, a fast-dissolving oral formulation of olanzapine has recently been developed [34,35] and has proven effective in patients requiring acute treatment.

The current IMPULSE study investigated treatment of acutely agitated patients including the short-term effectiveness and tolerability of olanzapine and other antipsychotic treatments (risperidone, haloperidol). The observational nature of this study enabled the inclusion of a highly agitated, heterogeneous group of patients, many of whom would have normally been excluded from randomised trials with strict in- and exclusion criteria. Thus, the results of this study are more representative of those patients generally encountered in an emergency treatment situation. However, as for all observational studies, the lack of randomisation and of independent, blinded assessments are clear limitations of this study. For this study in particular, it needs to be considered that patients receiving olanzapine treatment were over-represented in our population compared to the olanzapine market share, limiting the generalisability and representativity of our results. Furthermore, treatment groups were not fully comparable due to the lack of randomisation. Patients in the olanzapine cohort were less frequently pre-treated with antipsychotics than patients receiving no olanzapine, while patients receiving risperidone were more frequently pre-treated. Also, the olanzapine cohort tended to have lower CGI-A and CGI-SS ratings at baseline. These differences indicate that the olanzapine cohort may represent somewhat less severe patients at baseline.

Patients' psychiatric diagnoses and other background characteristics were quite varied, and should be reflecting the wider population of patients requiring immediate treatment for aggression: mean patient age was 40 years, about 63% were male, and large percentages abused psychoactive substances such as nicotine, alcohol, and/or illicit drugs. Large percentages of these patients were considered to be self-endangering or endangering to others, showed psychomotor agitation, or their behaviour required compulsory admission to a hospital or clinic. A majority of the patients in this study had an ICD-10 diagnosis of schizophrenia (F20–F29), and large proportions had mental disorders due to psychoactive substance abuse (F10–F19), mood disorders (F30–F39), or adult personality disorders (F60–F69).

About 70% of the patients (147 suffering from schizophrenia, 30 from manic episodes, and 213 with other diagnoses) in this study received at least one dose of olanzapine during the 5-day treatment and observation period, usually in combination with other APs. The remaining 30% were treated with APs other than olanzapine. The proportion of patients receiving antipsychotic polytherapy was much lower in the olanzapine than in the risperidone or haloperidol cohorts. However, these data may have been biased by the observational and non-randomised study design and the associated over-representation of olanzapine-treated patients.

Overall, about 70% of patients did receive benzodiazepines in addition to the AP medications. However, benzodiazepine use was numerically lower in the olanzapine-treated patients than in those receiving risperidone or haloperidol, and daily benzodiazepine doses were numerically lower in patients receiving haloperidol or olanzapine than in those receiving risperidone.

For the analyses used in this study, all treatment groups showed clear improvements of effectiveness measures even within the short observation time of this study. The PANSS-EC and CGI-aggression and CGI-suicidality scores improved to a comparable extent in the olanzapine, risperidone and haloperidol treatment groups. Nearly all patients were alert and active at the baseline measurement of the tranquilisation score. Patients in the olanzapine cohort were less sedated than the risperidone or haloperidol cohorts on all post-baseline days. This difference was most pronounced on day 2. This positive effect observed in the olanzapine cohort may have been caused by less frequent use of concomitant benzodiazepines, suggesting that olanzapine may achieve an effective immediate control of aggression without severe tranquilisation of the patient. Intake of lower amounts of benzodiazepines can be expected to reduce the incidence of adverse effects associated with benzodiazepines (e.g., sedation, ataxia, confusion, and respiratory depression), and reduce the incidence of adverse events caused by interactions between benzodiazepines and other antipsychotic medications. However, the limitations due to the observational and non-randomised study design need to be considered when evaluating these results.

## Conclusion

Immediate control of aggressive and impulsive behaviour can be effectively achieved through administration of antipsychotic medications, which are often administered without knowing the definite diagnosis in this specific setting. During the course of this short-term study, improvements in CGI-A and PANSS-EC were similar for olanzapine, risperidone and haloperidol treatment. The odds for receiving antipsychotic monotherapy were higher in patients treated with olanzapine. Compared to patients treated exclusively with other APs, those receiving olanzapine as antipsychotic monotherapy or part of combination treatment experienced a numerical reduction (not significant in exploratory statistical analysis) of benzodiazepine use and a positive effect on tranquilisation in the present non-randomised, naturalistic study. In patients receiving haloperidol, concomitant benzodiazepine use was significantly more frequent than in patients not treated with haloperidol. Given the huge number of patients requiring emergency inpatient or outpatient treatment for aggressive or impulsive behaviour, the use of atypical antipsychotics such as olanzapine may have the potential to reduce the medical burden for patients with this psychiatric condition.

## Abbreviations

AP: antipsychotic; CGI: Clinical Global Impression; CGI-A: CGI-aggression; CGI-SS: CGI-suicidality; EPS: extrapyramidal symptoms; Hal: haloperidol; ICD-10: International Classification of Diseases; LOCF: last observation carried forward; MedDRA: Medical Dictionary for Regulatory Activities; Olz: olanzapine; PANSS: Positive and Negative Syndrome Scale; Ris: risperidone.

## Competing interests

The study was funded and conducted by Lilly Deutschland GmbH, Bad Homburg, Germany. SW, AS, and TW are current employees of Eli Lilly and Company or Lilly Deutschland GmbH.

## Authors' contributions

SW, AS and TW participated in the design of the study and data interpretation and revised the manuscript. TW participated in the design of the study, prepared the protocol, participated in data interpretation, drafted the final study report, and reviewed the manuscript. AS provided statistical advice, participated in the statistical analyses and reviewed the manuscript. SW participated in data interpretation and reviewed the manuscript. All authors read and approved the final manuscript.

## Pre-publication history

The pre-publication history for this paper can be accessed here:



## References

[B1] Allen MH, Currier GW, Hughes DH, Reyes-Harde M, Docherty JP, Expert Consensus Panel for Behavioral Emergencies (2001). The Expert Consensus Guideline Series. Treatment of behavioural emergencies. Postgrad Med.

[B2] Allen MH, Currier GW, Carpenter D, Ross RW, Docherty JP, Expert Consensus Panel for Behavioral Emergencies 2005 (2005). The expert consensus guideline series. Treatment of behavioral emergencies 2005. J Psychiatr Pract.

[B3] Yildiz A, Sachs GS, Turgay A (2003). Pharmacological management of agitation in emergency settings. Emerg Med J.

[B4] Salzman C, Green AI, Rodriguez-Villa F, Jaskiw GI (1986). Benzodiazepines combined with neuroleptics for management of severe disruptive behavior. Psychosomatics.

[B5] Battaglia J, Moss S, Rush J, Kang J, Mendoza R, Leedom L, Dubin W, McGlynn C, Goodman L (1997). Haloperidol, lorazepam, or both for psychotic agitation? A multicenter, prospective, double-blind, emergency department study. Am J Emerg Med.

[B6] Tandon R, Jibson MD (2002). Extrapyramidal side effects of antipsychotic treatment: scope of problem and impact on outcome. Ann Clin Psychiatry.

[B7] Dilling H, Dittmann V (1990). Psychiatric diagnosis following the 10th revision of the International Classification of Diseases (ICD-10). Nervenarzt.

[B8] Lindenmayer JP (2000). The pathophysiology of agitation. J Clin Psychiatry.

[B9] Poser W, Poser S (1996). Medikamente – Missbrauch und Abhängigkeit. Entstehung – Verlauf – Behandlung.

[B10] Wright P, Meehan K, Birkett M, Lindborg SR, Taylor CC, Morris P, Breier A (2003). A comparison of the efficacy and safety of olanzapine versus haloperidol during transition from intramuscular to oral therapy. Clin Ther.

[B11] Kay SR, Fizbein A, Opler LA (1987). The positive and negative syndrome scale (PANSS) for schizophrenia. Schizophr Bull.

[B12] Baker RW, Kinon BJ, Maguire GA, Liu H, Hill AL (2003). Effectiveness of rapid initial dose escalation of up to forty milligrams per day of oral olanzapine in acute agitation. J Clin Psychopharmacol.

[B13] Guy W (1976). ECDEU Assessment Manual for Psychopharmacology, revised. DHEW Pub No (ADM) 76-338.

[B14] Guy W, Guy W (1976). Clinical global impression. ECDEU assessment manual for psychopharmacology, revised.

[B15] Sharif ZA, Raza A, Ratakonda SS (2000). Comparative efficacy of risperidone and clozapine in the treatment of patients with refractory schizophrenia or schizoaffective disorder: a retrospective analysis. J Clin Psychiatry.

[B16] Sival RC, Duivenvoorden HJ, Jansen PA, Haffmans PM, Duursma SA, Eikelenboom P (2004). Sodium valproate in aggressive behaviour in dementia: a twelve-week open label follow-up study. Int J Geriatr Psychiatry.

[B17] MacMillan CM, Korndörfer SR, Rao S, Fleisher CA, Mezzacappa E, Gonzalez-Heydrich J (2006). A comparison of divalproex and oxcarbazepine in aggressive youth with bipolar disorder. J Psychiatr Pract.

[B18] Lindenmayer JP, Czobor P, Alphs L, Nathan AM, Anand R, Islam Z, Chou JC, InterSePT Study Group (2003). The InterSePT scale for suicidal thinking reliability and validity. Schizophr Res.

[B19] Karagianis JL, Dawe IC, Thakur A, Bégin S, Raskin J, Roychowdhury SM (2001). Rapid tranquilization with olanzapine in acute psychosis: a case series. J Clin Psychiatry.

[B20] Hazlett SB, McCarthy ML, Londner MS, Onyike CU (2004). Epidemiology of adult psychiatric visits to US emergency departments. Acad Emerg Med.

[B21] Pajonk FG, Grunberg KA, Paschen HR, Moecke H, Arbeitsgruppe Psychiatrie und Rettungswesen (2001). Psychiatric emergencies in the physician-based system of a German city. Fortschr Neurol Psychiatr.

[B22] Lindenmayer JP, Khan A (2004). Pharmacological treatment strategies for schizophrenia. Expert Rev Neurother.

[B23] Pajonk FG, Fleiter B (2003). Psychopharmacological treatment in the pre-clinical emergency medicine. Anaesthesist.

[B24] Marco CA, Vaughan J (2005). Emergency management of agitation in schizophrenia. Am J Emerg Med.

[B25] Nestor PG (2002). Mental disorder and violence: personality dimensions and clinical features. Am J Psychiatry.

[B26] Stedman HJ, Mulvey EP, Monahan J (1998). Violence by people discharged from acute psychiatric facilities and by others in the same neighbourhoods. Arch Gen Psychiatry.

[B27] TREC Collaborative Group (2003). Rapid tranquillisation for agitated patients in emergency psychiatric rooms: a randomised trial of midazolam versus haloperidol plus promethazine. BMJ.

[B28] Alexander J, Tharyan P, Adams C, John T, Mol C, Philip J (2004). Rapid tranquillisation of violent or agitated patients in a psychiatric emergency setting. Pragmatic randomised trial of intramuscular lorazepam v. haloperidol plus promethazine. Br J Psychiatry.

[B29] Krakowski MI, Czobor P, Citrome L, Bark N, Cooper TB (2006). Atypical antipsychotic agents in the treatment of violent patients with schizophrenia and schizoaffective disorder. Arch Gen Psychiatry.

[B30] Humble F, Berk M (2003). Pharmacological management of aggression and violence. Hum Psychopharmacol.

[B31] Meehan K, Zhang F, David S, Tohen M, Janicak P, Small J, Koch M, Rizk R, Walker D, Tran P, Breier A (2001). A double-blind, randomized comparison of the efficacy and safety of intramuscular injections of olanzapine, lorazepam, or placebo, in treating acutely agitated patients diagnosed with bipolar mania. J Clin Psychopharmacol.

[B32] Meehan KM, Wang H, David SR, Nisivoccia JR, Jones B, Beasley CM, Feldman PD, Mintzer JE, Beckett LM, Breier A (2002). Comparison of rapidly acting intramuscular olanzapine, lorazepam, and placebo: a double blind, randomised study in acutely agitated patients with dementia. Neuropsychopharmacology.

[B33] Breier A, Meehan K, Birkett M, David S, Ferchland I, Sutton V, Taylor CC, Palmer R, Dossenbach M, Kiesler G, Brook S, Wright P (2002). A double-blind, placebo-controlled dose-response comparison of intramuscular olanzapine and haloperidol in the treatment of acute agitation in schizophrenia. Arch Gen Psychiatry.

[B34] Czekalla J, Wagner T, Schacht A, Kluge M, Kinon B (2007). Effectiveness and medication acceptance of olanzapine disintegrating tablets compared to standard olanzapine tablets in acutely treated psychiatric patients. Patient Preference and Adherence.

[B35] Czekalla J, Linder P, Wagner T Improvement of suicidal ideation and medication acceptance under treatment with orally disintegrating and coated olanzapine tablets – results from a prospective multi-center study of acutely ill psychiatric inpatients [Abstract]. Eleventh Biennal Winter Workshop on Schizophrenia, February 7–14, 2004, Davos, Switzerland.

